# Synergistic Effects of Slurry Concentration and Binder Reactivity on the Hydraulic Transport of Unclassified Tailings Backfill

**DOI:** 10.3390/ma19040768

**Published:** 2026-02-16

**Authors:** Ning Yang, Renze Ou, Zirui Li, Daoyuan Sun, Hongwei Wang, Qi Liu, Mingdong Tang, Xiaohui Li

**Affiliations:** 1 School of Resources and Safety Engineering, Central South University, Changsha 410083, China; 225502039@csu.edu.cn (N.Y.); 255502047@csu.edu.cn (R.O.); 255512022@csu.edu.cn (Z.L.); whuwhw@whu.edu.cn (H.W.); 2 Changsha Institute of Mine Research Co., Ltd., China Minmetals Corporation, Beijing 100010, China; liuqicsy@163.com (Q.L.); tangmd6@minmetals.com (M.T.); lixiaohui@cumt.edu.cn (X.L.)

**Keywords:** unclassified tailings cemented backfill, backfill strength, L-shaped pipeline simulation test, deep mining

## Abstract

To address the safety and environmental challenges associated with deep mining, this study investigates the rheological behaviors and pipeline transport characteristics of cemented paste backfill (CPB) using unclassified tailings from a lead–zinc mine. Through the characterization of basic physicochemical properties—including chemical composition, particle size distribution, and specific surface area—combined with L-shaped pipeline simulation tests, the effects of slurry concentration and pipe diameter on rheological parameters and transport resistance were quantitatively analyzed. Furthermore, the mechanical performance and cost-effectiveness of four different cementitious binders were evaluated to identify the optimal material. The results indicate that the unclassified tailings possess a favorable particle size distribution with a significant fine-particle filling effect, making them suitable as backfill aggregates. Slurry concentration was identified as the critical factor influencing rheological performance; a concentration range of 68% to 72% was determined to be optimal, exhibiting superior fluidity and low pipeline resistance conducive to gravity flow. Additionally, increasing the pipe diameter was found to effectively reduce transport difficulty. Based on a comprehensive technical and economic analysis, Kunlun Mountain PO42.5 cement was selected as the optimal binder, achieving the required backfill strength with controlled costs. This study provides a theoretical basis and practical engineering guidance for the design and optimization of deep-well backfill pipeline systems.

## 1. Introduction

With the continuous depletion of shallow mineral resources, Xie et al. [1] and Fairhurst [2] pointed out that the global mining industry has strategically shifted towards deep mining to meet the increasing demand for mineral resources. However, Yuan [3] emphasized that deep mining operations are characterized by high ground stress and complex mechanical environments, posing severe risks to safety. Simultaneously, Adiansyah et al. [4] noted that the large-scale accumulation of surface tailings has caused significant environmental degradation. To address these challenges, Wu et al. [5] promoted Cemented Paste Backfill (CPB) technology as a pillar of “Green Mining” in China. Qi and Fourie [6] further elaborated that this method effectively mitigates surface subsidence and controls rock strata movement.

The success of a CPB system relies heavily on rheology. Yu et al. [7] and Boger et al. [8] established that the rheological properties of the slurry dictate pumpability and transport efficiency. Recent studies by Lan et al. [9] in engineering highlighted that these parameters are highly sensitive to solid concentration. Specifically, Wu et al. [10] investigated the coupled rheological behavior of CPB and highlighted that the yield stress is highly sensitive to solid concentration, increasing exponentially due to particle interlocking. Cheng et al. [11] confirmed that this phenomenon is driven by the transition from lubrication to frictional contact. Regarding particle size, Monteiro et al. [12] investigated the mechanical behavior of hybrid fiber-reinforced composites and found that fiber hybridization significantly enhances post-cracking strength. Fall et al. [13] confirmed that increasing tailings fineness effectively enhances the suspension capability and stability of the mix, whereas Deng et al. [14] experimentally demonstrated that the presence of ultra-fine particles significantly increases the yield stress and plastic viscosity of the paste. Additionally, Xiu et al. [15] systematically investigated the effects of solid content and binder dosage, demonstrating that solid content is the dominant factor determining the rheological parameters of the paste.

Pipeline transport stability is another focus. Zhang et al. [16] stressed its criticality for deep-well backfilling. Belem and Benzaazoua [17] systematically reviewed the critical parameters for pipeline transport stability and highlighted that poor rheological control is a primary cause of blockage, while Chen et al. [18] employed Computational Fluid Dynamics (CFD) to simulate wall shear stress. However, Ren et al. [19] highlighted that the rheological behavior of paste is highly complex and sensitive to external conditions, which often leads to deviations in theoretical predictions. Consequently, Qi et al. [20] and Zhou et al. [21] argued that optimizing transport parameters based on site-specific material properties is vital for engineering design.

Binder selection also plays a pivotal role. Tariq and Yanful [22] reviewed various binders, noting that while they provide strength, Fall et al. [23] found that their early-age hydration kinetics significantly affect initial flowability. From a sustainability perspective, Tost et al. [24] analyzed the environmental pressures of mining, highlighting the need for low-carbon solutions such as binder optimization. Ghirian and Fall [25] revealed that coupled thermo-hydro-chemical effects can accelerate hydration and structural buildup. To reduce costs, Cihangir et al. [26] and Kou et al. [27] investigated alkali-activated slag, reporting promising rheological properties. However, Ercikdi et al. [28] investigated alkali-activated slag, demonstrating its potential to replace cement and improve mechanical performance. Furthermore, Jiang et al. [29] investigated alkali-activated slag, demonstrating its potential to replace cement and improve mechanical performance, and Li and Fall [30] highlighted durability issues such as sulfate attack and self-desiccation.

Recent advancements have focused on functional improvements. Yin et al. [31] introduced expansive agents to improve roof contact, and Mangane et al. [32] highlighted that incorporating superplasticizers can significantly improve the workability and mechanical properties of the paste. Cao et al. [33] introduced fiber reinforcement, demonstrating its effectiveness in enhancing the toughness and microstructure of the backfill. Wickland et al. [34] proposed the co-disposal of waste rock and tailings to optimize the mechanical stability and void ratio of the backfill matrix. Fundamental research by Yilmaz et al. [35] on consolidation and Fall et al. [36] emphasized that curing temperature significantly influences the hydration process and strength development, highlighting the difference between lab and in-situ conditions. Moreover, Yilmaz et al. [37] demonstrated that curing under pressure leads to higher strength development compared to standard atmospheric curing, and Ghirian and Fall investigated the coupled thermo-hydro-mechanical-chemical behavior, revealing the complex interactions between mechanical stress, chemical curing, and microstructure evolution. Finally, Grabinsky et al. [38] reported significant discrepancies between laboratory results and in situ performance, highlighting the critical need for field validation. Belem and Benzaazoua established a comprehensive design framework to bridge the gap between laboratory characterization and industrial application.

## 2. Multi-Dimensional Testing of Backfill Materials

### 2.1. Test Materials

Unclassified Tailings: The aggregate used in this experiment was unclassified tailings collected from the underflow of the flotation process at the lead–zinc mine. To ensure the homogeneity and representativeness of the samples used in laboratory tests, the fresh tailings slurry was subjected to precipitation, natural air-drying, and mechanical disaggregation to remove agglomerates. The prepared tailings samples were sealed in moisture-proof bags for subsequent characterization and slurry preparation.

Cementitious Binders: To investigate the influence of binder composition and reactivity on slurry rheology, four types of cement were selected: Kunlun Mountain PO42.5, Baishushan PO42.5, Jinyuan PC32.5R, and Baishushan PC32.5R. All binders conform to the National Standard GB 175-2007. Specifically, “PO” represents Ordinary Portland Cement with a clinker content exceeding 80%, chosen for its high strength potential. “PC” represents Composite Portland Cement, which contains a higher proportion of supplementary cementitious materials. The suffix “R” denotes rapid-hardening type, selected to evaluate its effect on the initial structural buildup of the backfill slurry.

### 2.2. Characterization of Basic Properties of Unclassified Tailings

#### 2.2.1. Basic Chemical Parameters

The chemical composition of the unclassified tailings was determined using X-ray fluorescence (XRF) spectrometry, the results are shown in Table 1, focusing on elements significantly influencing the cementation process and backfill strength. The inorganic residues critically influence the backfill properties through coupled chemical and physical mechanisms. Chemically, the silicon content is of particular significance. It indicates the presence of stable crystalline silicate minerals (likely quartz) which possess high hardness and elastic modulus. These minerals act as a rigid skeletal framework to bear compressive loads and, due to their chemical inertness, provide enhanced resistance against potential acidic erosion in the mine environment. Meanwhile, the low sulfur content prevents internal sulfate attack and expansive mineral formation, ensuring volume stability.

#### 2.2.2. Basic Physical Parameters

Key physical indices of the unclassified tailings were measured, and the results are listed in Table 2.

### 2.3. Determination of Particle Size Distribution of Unclassified Tailings

The particle size of tailings significantly impacts mine backfilling and is related to the cementation performance of the backfill. Laser diffraction was used to analyze the particle size distribution of the unclassified tailings. Key parameters analyzed include the median diameter and the uniformity coefficient.

**Figure 1:**
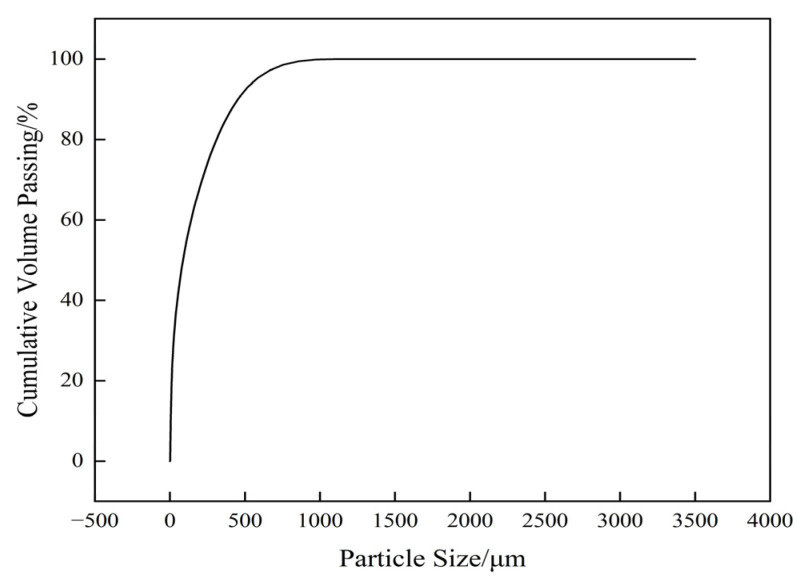
Tailings particle size distribution statistics.

The particle size distribution of the lead–zinc mine tailings was analyzed using a Mastersizer laser diffraction analyzer. As shown in Figure 1, the characteristic particle diameters were determined to be d10 = 5.43 µm, d50 = 87.3 µm, d60 = 144 µm, and d90 = 453 µm.

The particle uniformity coefficient is

(1) $$C_{u}=\frac{d_{60}}{d_{10}}\geq 5$$

where d_10_ is the sieve diameter (µm) through which 10% of the loose material can pass; similarly, d_50_, d_60_, and d_90_ represent the sieve diameters for 50%, 60%, and 90% passing, respectively.

A larger $C_{u}$ indicates non-uniform particle composition, which is beneficial as fine particles can fill voids between coarse particles, forming a denser backfill structure. Furthermore, the content of fine particles (−20 µm) exceeds 15%, indicating a favorable particle size distribution.

The specific surface area of the lead–zinc mine tailings was analyzed using a US Quantachrome Monosorb direct-reading specific surface area analyzer, and the result was 359.7 m^2^/kg.

### 2.4. Experimental Repeatability and Data Analysis

To ensure the statistical significance and reproducibility of the rheological parameter calculation model, all L-shaped pipeline transport tests were performed in triplicate under identical conditions. The data presented represent the mean values, with error bars indicating the standard deviation. A statistical analysis of the raw experimental data indicated that the relative standard deviation was consistently controlled within 5%. This low variance confirms that the experimental inputs for the calculation model possess high statistical reliability, minimizing the influence of random experimental errors on the derived rheological parameters.

## 3. Experimental Results Analysis and Discussion

### 3.1. Basic Principle of Rheological Parameter Calculation Model

Based on the Bingham fluid equation and considering a uniform flow velocity V across the pipe cross-section, the following equation can be derived from the Bernoulli equation:

(2) $$\frac{8V}{D}=(\tau /\eta )\left[1-\frac{4}{3}\left(\frac{\tau_{0}}{\tau }\right)+\frac{1}{3}{\left(\frac{\tau_{0}}{\tau }\right)}^{4}\right]$$

It is generally accepted that the high-order term of $\frac{\tau_{0}}{\tau }$ is very small and can be neglected, yielding an approximate expression for wall shear stress:

(3) $$\tau =\frac{4}{3}\tau_{0}+\frac{8\eta V}{D}$$

where: V—flow velocity of backfill slurry, m/s; D—pipe diameter, m; $\tau_{0}$—yield shear stress, Pa; τ—pipe wall shear stress, Pa; η—viscosity coefficient, Pa·S.

The stress state of the flowing backfill slurry is shown in Figure 1.

According to the law of energy conservation, the following formula applies:

(4) $$P_{0}+P_{g}=P_{l}+P^{\prime }$$

where $P_{0}$ is the inlet pressure, calculated using

(5) $$P_{0}=\gamma h^{\prime }$$

$P_{g}$ is the gravitational static pressure due to the slurry column, calculated using

(6) $$P_{g}=\gamma h$$

$P_{l}$ is the frictional head loss, calculated using

(7) $$P_{l}=P_{straight}+P_{local}=\frac{4\tau (h+L)}{D}+P_{local}$$

The local loss $P_{local}$ includes losses from bends, joints, etc. Here, it is taken as 10% of the straight pipe loss.

$P^{\prime }$ is the exit pressure loss, calculated using

(8) $$P^{\prime }=\gamma \frac{V^{2}}{2g}$$

where γ, *D*, L, h, h’, V, $\tau_{0}$, η, and g indicate the Bulk density of slurry (kN/m^3^), test pipe diameter (m), vertical length of the test pipe (m), height of the hopper’s center of mass relative to the pipe inlet (m), slurry velocity in the test pipe (m/s), calculated shear stress for the test condition (Pa), viscosity coefficient (Pa), and gravitational acceleration (9.8 m/s^2^), respectively.

Substituting into Formula (1) yields

(9) $$\frac{\gamma D}{4}(h+h^{\prime })=1.10\tau (h+L)+\gamma \frac{V^{2}\cdot D}{8g}$$

As the test proceeds, the slurry level in the hopper drops, and the flow velocity decreases until it reaches 0. When flow finally stops, the height of the slurry column in the vertical pipe is h0, where the slurry’s self-weight pressure balances the static frictional resistance in the pipe. The yield shear stress of the slurry can then be calculated as follows:

(10) $$\tau_{0}=\frac{\gamma \cdot h_{0}D}{4(h_{0}+L)}$$

where h_0_ is the height of the slurry column (m) in the vertical pipe under static conditions.

During the tests, unclassified tailings backfill slurries with different concentrations were prepared. Their slump and bulk density were measured, and the flow velocity V in the pipe was determined. Using Equations (9) and (10), the corresponding τ and${\;\tau }_{0}$ can be calculated. Subsequently, the viscosity coefficient η of the slurry can be calculated from Equation (3), i.e.,

(11) $$\eta =\frac{(3\tau -4\tau_{0})\cdot D}{24V}$$

For this experimental setup, h = 1.2, h’ = 0.18 m, D = 0.06 m, and L = 2.06 m.

**Figure 2:**
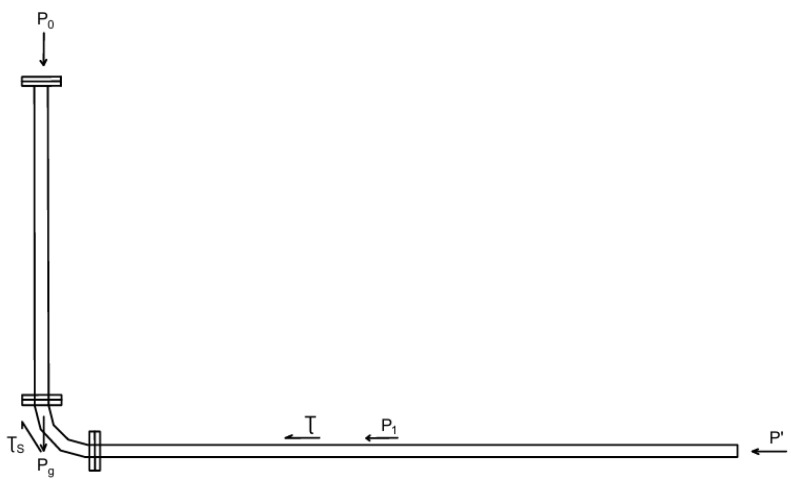
Stress state diagram of flowing backfill slurry.

**Figure 3:**
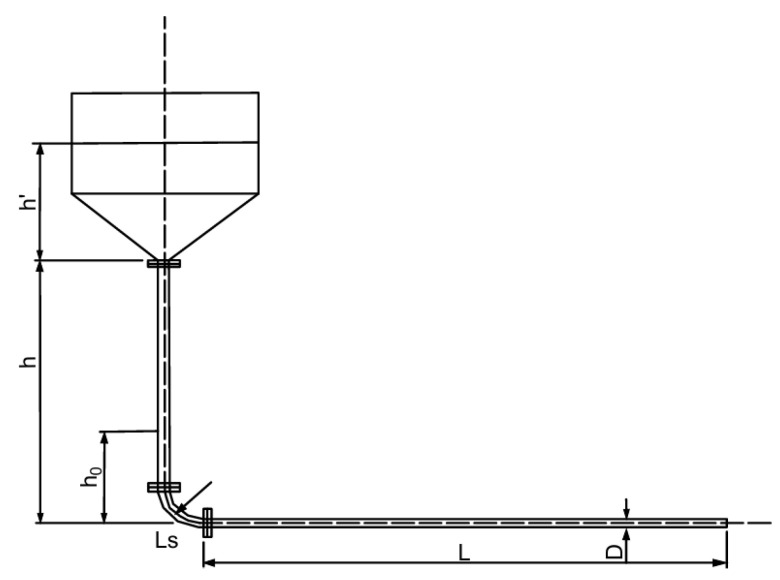
Schematic diagram of gravity flow transport test setup structure and dimensions.

In the laboratory, an L-shaped pipeline gravity flow transport test setup, as shown in Figure 2 and Figure 3, were used for measurement.

### 3.2. Slurry Flow Velocity

Slurry flow velocity V (m/s):

(12) $$V=\frac{Q}{3600\times \frac{\pi }{4}D^{2}}$$

where Q—flow rate of backfill slurry, m^3^/h.

### 3.3. Flow Resistance

Based on the rheological parameters of unclassified tailings backfill slurries at different concentrations, the transport resistance for industrial-scale conditions with varying slurry concentrations, flow rates, and pipe inner diameters can be calculated using the following formula.

Flow resistance per unit pipe length i (Pa/m):

(13) $$i=\frac{16\tau_{0}}{3D}+\frac{32\eta V}{D^{2}}$$

where i—flow resistance of slurry inside the pipe, Pa/m; V—slurry velocity in the backfill pipeline under industrial conditions, m/s; D—inner diameter of the backfill pipeline, m.

### 3.4. Transport Gradient and Gravity Flow Judgment

For a mine backfill pipeline network under gravity flow conditions, if the vertical pipe height is H and the horizontal pipe length is L, the principle of energy conservation yields

(14) $$\gamma H=i(H+L)+P_{local}+\gamma \frac{V^{2}}{2g}$$

$P_{local}$ represents local pipeline resistance. Taking the sum of local resistance and exit loss as 15% of the frictional head loss, the above equation becomes

(15) $$\frac{H+L}{H}=\frac{\gamma }{1.15i}$$

Here, (H + L)/L is the ratio of total pipeline length to vertical height, known as the backfill gradient.

### 3.5. Test Results and Analysis of Unclassified Tailings Slurry

The static yield stress is calculated based on the equilibrium height of the slurry column after it naturally stops flowing. In this experimental setup, the measurement is taken at the moment the flow stops.

To establish the rheological baseline of total tailings slurry under non-cementitious conditions, L-shaped pipeline transportation tests were first carried out on total tailings slurry with five mass concentrations ranging from 66% to 74%.

**Figure 4:**
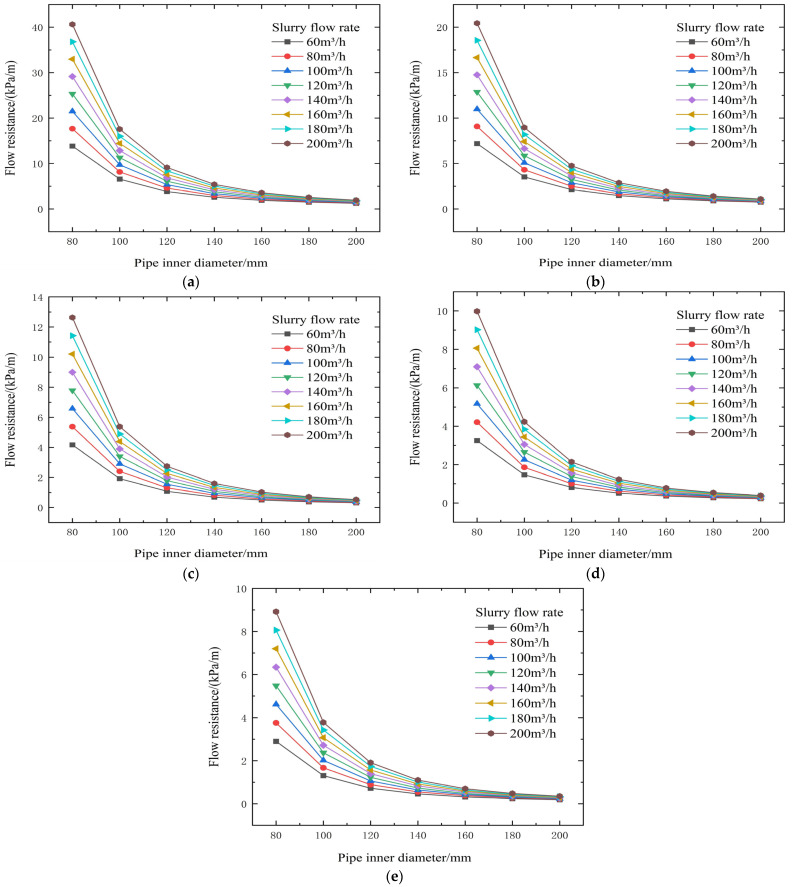
Calculated flow resistance results for different concentrations, pipe diameters, and flow rates (no binder). (**a**) 74% slurry concentration, (**b**) 72% slurry concentration, (**c**) 70% slurry concentration, (**d**) 68% slurry concentration, and (**e**) 66% slurry concentration.

**Figure 5:**
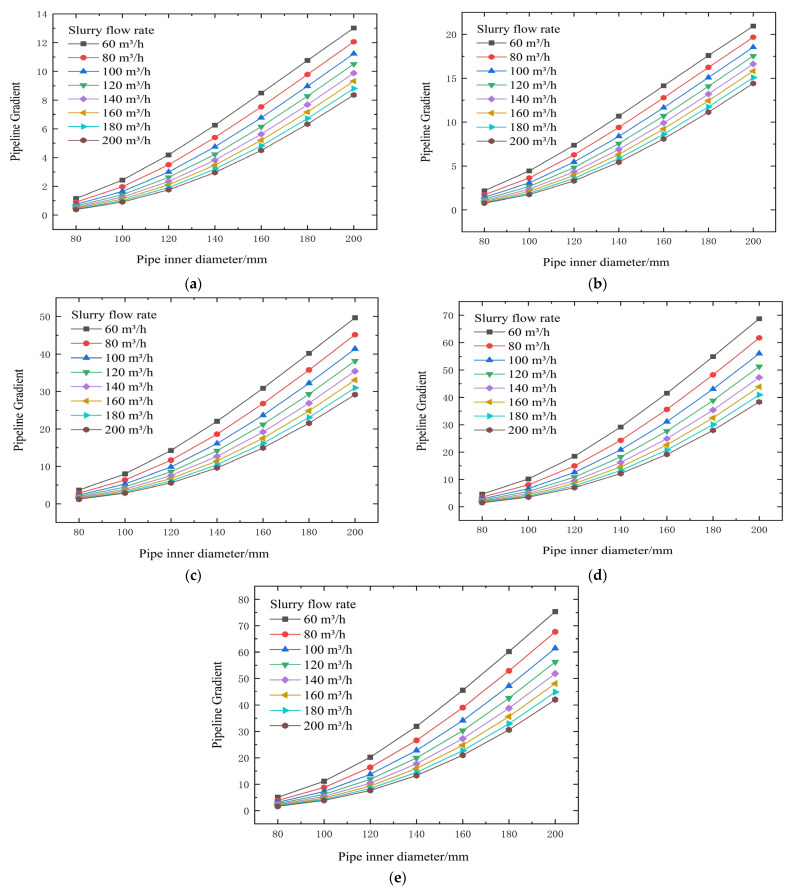
Calculated backfill gradient for different concentrations, pipe diameters, and flow rates (no binder). (**a**) 74% slurry concentration, (**b**) 72% slurry concentration, (**c**) 70% slurry concentration, (**d**) 68% slurry concentration, and (**e**) 66% slurry concentration.

From the test results and theoretical analysis in Table 3 and Figure 4 and Figure 5, experimental data and theoretical analysis show that slurry concentration is the core factor determining its rheological properties. When the concentration drops below 72%, both the yield stress ($\tau_{0}$) and the viscosity coefficient (η) decrease stepwise, significantly reducing transport resistance. Simultaneously, pipe diameter is a key engineering variable for controlling resistance. Theoretical formulas show that resistance is related to a negative power of the diameter D; increasing the pipe diameter can be extremely effective in reducing resistance.

To verify the applicability of the rheological models used in this study, the flow regimes for the reported cases were examined by calculating the Reynolds number using the equation Re=ρVD/η. Based on the data in Table 3, with slurry velocities ranging from 0.68 to 3.01 m/s and viscosities from 0.156 to 0.693 Pa·s, the calculated Reynolds numbers are approximately 111, 489, 1221, 1732, and 1990 for concentrations of 74%, 72%, 70%, 68%, and 66%, respectively. In all tested cases, the Reynolds numbers remain below the critical threshold for pipe flow (typically $Re_{c}\approx 2300$), indicating that the slurry flow within the experimental L-shaped pipeline remains strictly in the laminar regime. Consequently, the head-loss relations based on the Bingham plastic fluid model (Equations (2) and (13)) are valid for the analysis of these experimental results, as no turbulent behavior was observed. Consequently, the head-loss relations based on the Bingham plastic fluid model are valid for the analysis of the unclassified tailings slurry.

To systematically compare the effects of different cementitious materials on slurry transportation performance, a series of tests were conducted on four types of cement under a fixed cement–sand ratio (1:10). Firstly, tests were carried out on the slurry mixed with Jinyuan PC32.5R cement. It is noted that for all cemented backfill slurries tested in the subsequent sections (Table 4, Table 5, Table 6 and Table 7), particularly within the optimal concentration range (68–72%), the calculated Reynolds numbers consistently remained within the laminar flow regime Re<2300, ensuring the continued applicability of the rheological models.

**Figure 6:**
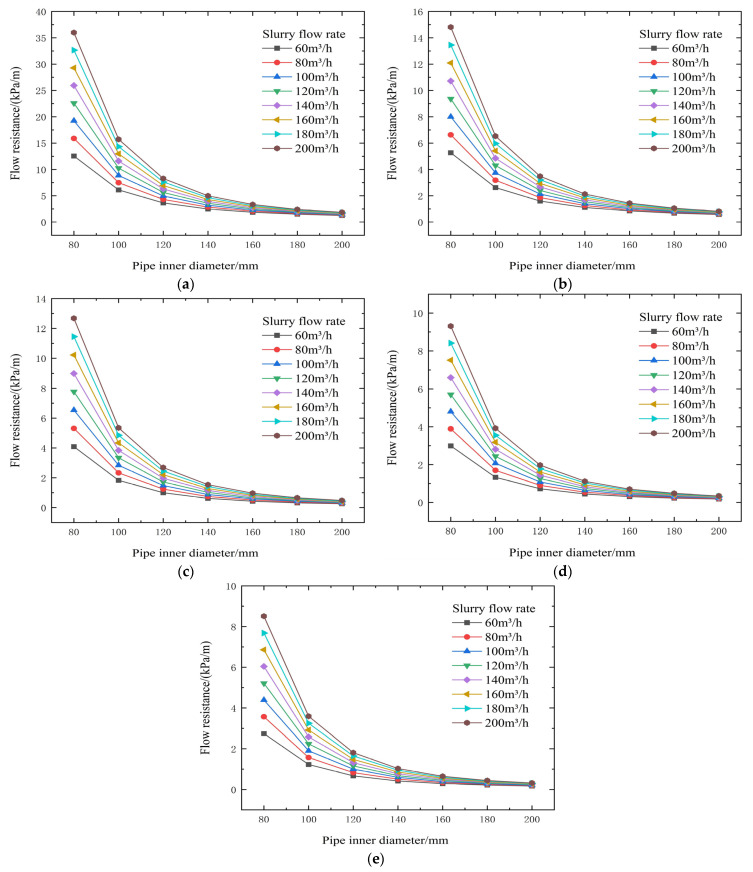
Calculated flow resistance results for different concentrations, pipe diameters, and flow rates (cement-to-tailings ratio 1:10, unit: kPa/m). (**a**) 74% slurry concentration, (**b**) 72% slurry concentration, (**c**) 70% slurry concentration, (**d**) 68% slurry concentration, and (**e**) 66% slurry concentration.

**Figure 7:**
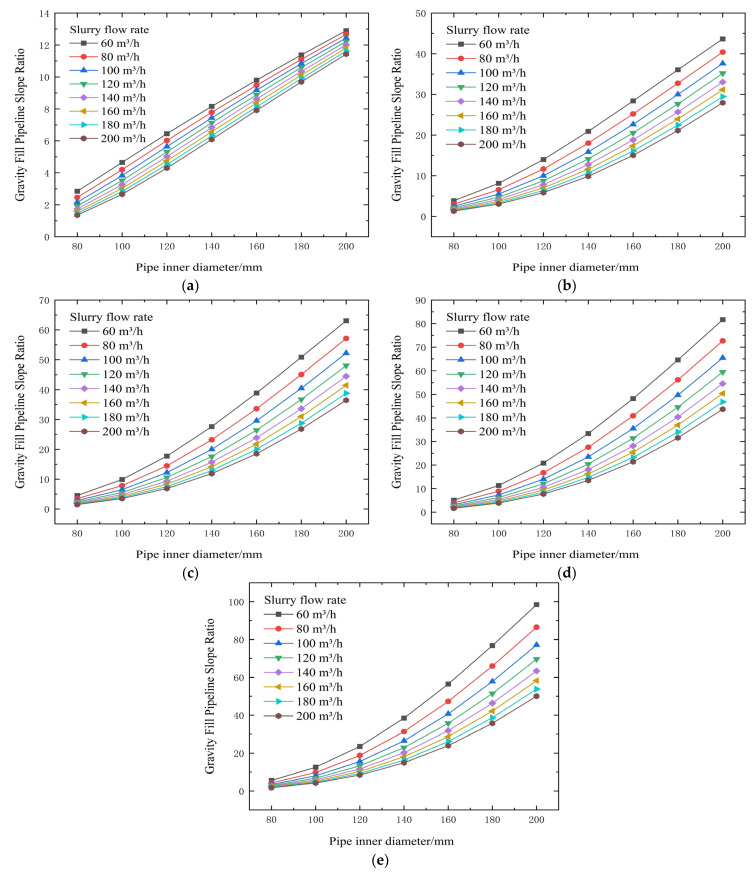
Calculated gravity backfill gradient for different concentrations, pipe diameters, and flow rates. (**a**) 74% slurry concentration, (**b**) 72% slurry concentration, (**c**) 70% slurry concentration, (**d**) 68% slurry concentration, and (**e**) 66% slurry concentration.

From the test results and theoretical analysis in Table 4 and Figure 6 and Figure 7, the yield shear stress τ is positively correlated with slurry concentration. When concentration decreases from 74% to 66%, τ significantly decreases, which is beneficial for reducing resistance. The viscosity coefficient η decreases with decreasing concentration. When concentration is <72%, η is generally below 0.428 Pa·s, resulting in lower transport resistance. Increasing flow velocity V leads to increased resistance. Pipe diameter D is a critical factor affecting resistance. Increasing the pipe diameter can substantially reduce resistance. For example, with the same cement-to-tailings ratio and concentration at a flow rate of 100 m^3^/h, increasing the pipe diameter from 80 mm to 140 mm reduces resistance from 8.00 kPa/m to 1.40 kPa/m, and increases the backfill gradient from 1.89 to 10.80.

To further investigate the effects of cement grade and activity on the pipeline transportation of slurry, tests were conducted on the slurry mixed with Kunlun Mountain PO42.5 cement. This series of tests aimed to obtain the rheological performance of high-grade cement under the same cement–sand ratio and evaluate the comprehensive effects of cement properties on transportation resistance and filling gradient by comparing the data with that of low-grade cement and cement of different brands, thereby to providing a basis for engineering material selection.

**Figure 8:**
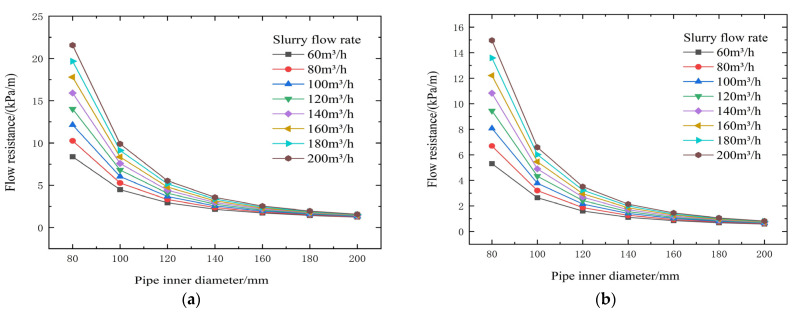

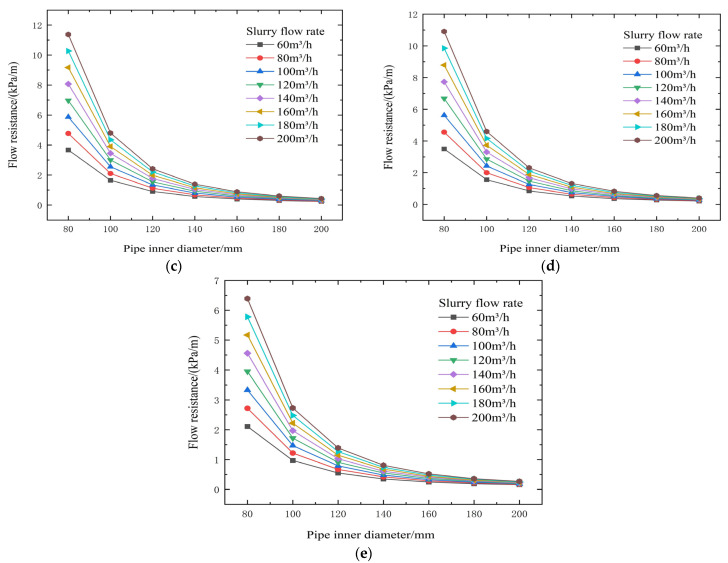
Calculated flow resistance results for different concentrations, pipe diameters, and flow rates (cement-to-tailings ratio 1:10, unit: kPa/m). (**a**) 74% slurry concentration, (**b**) 72% slurry concentration, (**c**) 70% slurry concentration, (**d**) 68% slurry concentration, and (**e**) 66% slurry concentration.

**Figure 9:**
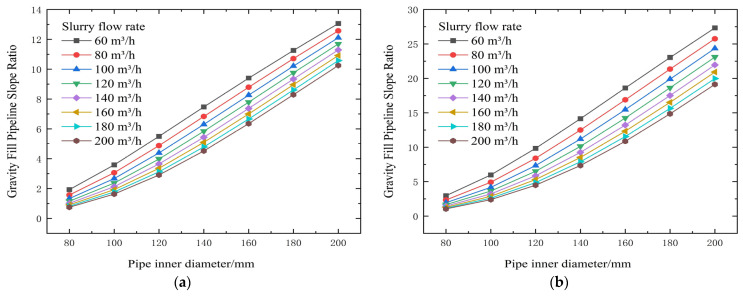

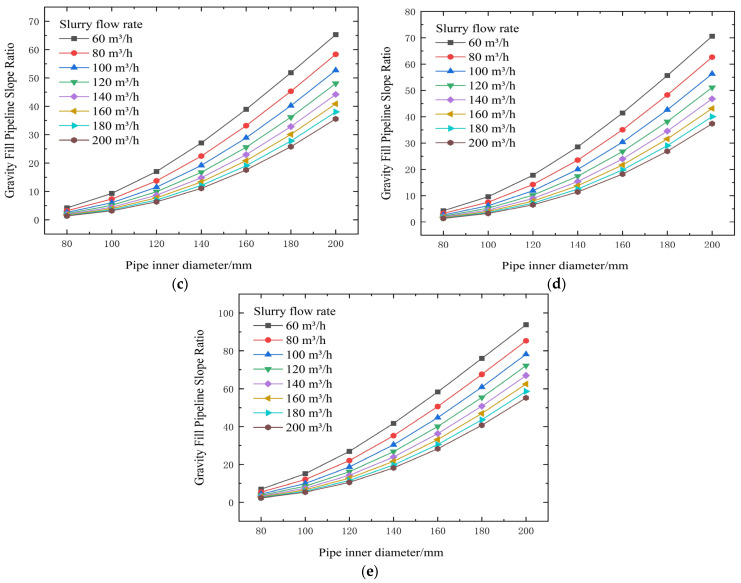
Calculated gravity backfill gradient for different concentrations, pipe diameters, and flow rates. (**a**) 74% slurry concentration, (**b**) 72% slurry concentration, (**c**) 70% slurry concentration, (**d**) 68% slurry concentration, and (**e**) 66% slurry concentration.

From the test results and theoretical analysis in Table 5 and Figure 8 and Figure 9, both yield shear stress and viscosity coefficient increase significantly with increasing slurry concentration. When the concentration is controlled below 72%, both parameters decrease substantially. Yield stress can drop from 47.87 Pa to 5.05 Pa (example from context), effectively reducing transport resistance. Increasing flow velocity elevates resistance; for a 120 mm pipe diameter, increasing velocity from 1.474 m/s to 2.947 m/s raises resistance from 2.01 kPa/m to 3.03 kPa/m. Conversely, enlarging the pipe diameter can drastically reduce resistance. For example, at a flow rate of 100 m^3^/h, increasing the pipe diameter from 80 mm to 140 mm can sharply reduce resistance from 10.09 kPa/m to 1.77 kPa/m, correspondingly increasing the backfill gradient from 1.56 to 8.88.

Subsequent tests were conducted on Baishan PC32.5R cement. This part aimed to obtain its rheological response under the same concentration and mix ratio conditions, with particular focus on the performance differences between this cement and Jinyuan cement of the same grade in terms of slurry viscosity, yield stress and other aspects, thereby providing a multi-dimensional comparison basis for cement selection.

**Figure 10:**
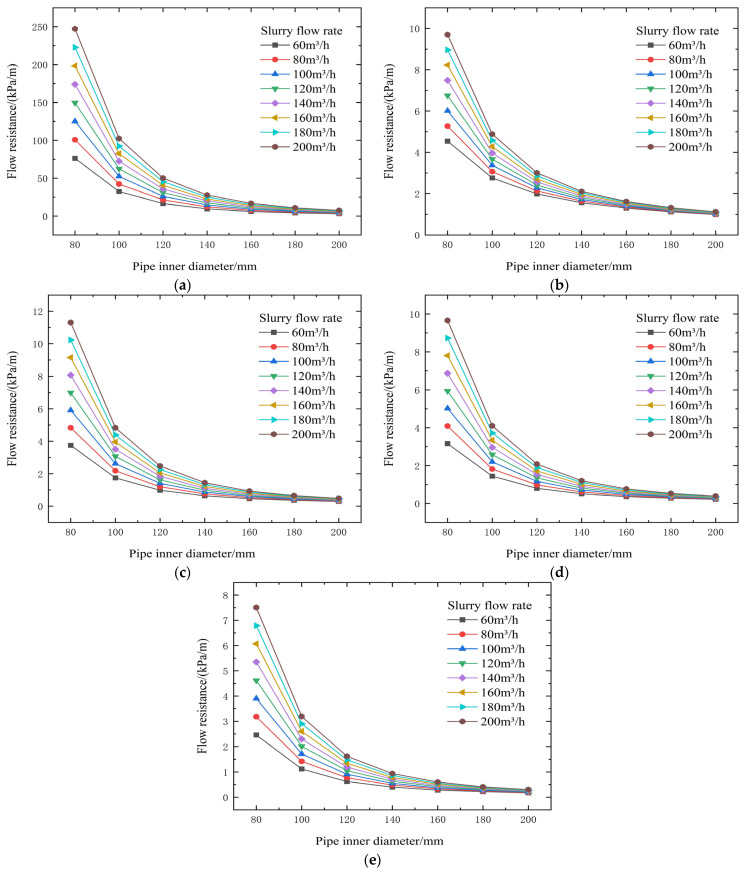
Calculated flow resistance results for different concentrations, pipe diameters, and flow rates (cement-to-tailings ratio 1:10, unit: kPa/m). (**a**) 74% slurry concentration, (**b**) 72% slurry concentration, (**c**) 70% slurry concentration, (**d**) 68% slurry concentration, and (**e**) 66% slurry concentration).

**Figure 11:**
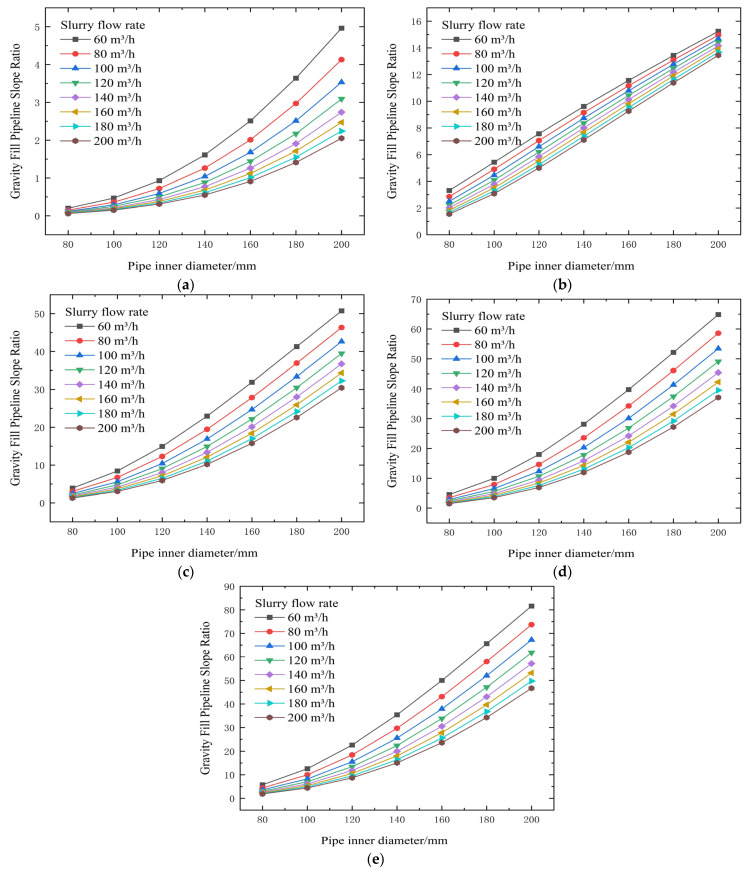
Calculated gravity backfill gradient for different concentrations, pipe diameters, and flow rates. (**a**) 74% slurry concentration, (**b**) 72% slurry concentration, (**c**) 70% slurry concentration, (**d**) 68% slurry concentration, and (**e**) 66% slurry concentration.

From the test results and theoretical analysis in Table 6 and Figure 10 and Figure 11, tests show that when concentration decreases from 74% to 66%, yield stress decreases significantly from 50.57 Pa to 4.42 Pa (example from context), and the viscosity coefficient also drops substantially after concentration falls below 72%, from 4.417 Pa·s to 0.134 Pa·s, thereby reducing transport resistance. Increasing flow velocity raises resistance.

Further tests were conducted to evaluate the transportation performance of the slurry mixed with Baishan PO42.5 cement. This test aimed to complete a systematic dataset for the four types of cement under identical test conditions, provide comprehensive data support for the subsequent holistic analysis of the coupling relationship among cement type, slurry concentration, and pipeline transportation performance, and ultimately facilitate the optimal selection of cementitious materials for engineering.

**Figure 12:**
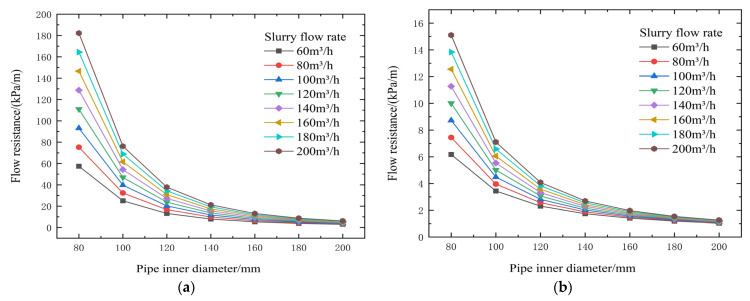

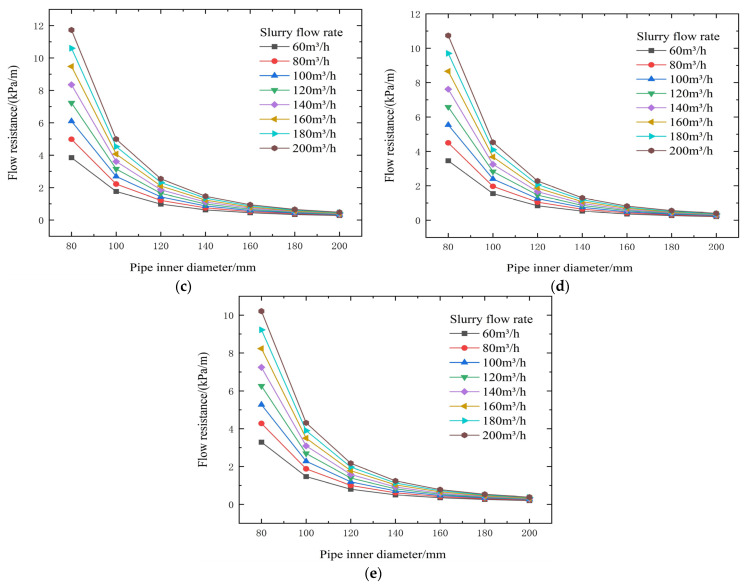
Calculated flow resistance results for different concentrations, pipe diameters, and flow rates (cement-to-tailings ratio 1:10, unit: kPa/m). (**a**) 74% slurry concentration, (**b**) 72% slurry concentration, (**c**) 70% slurry concentration, (**d**) 68% slurry concentration, and (**e**) 66% slurry concentration).

**Figure 13:**
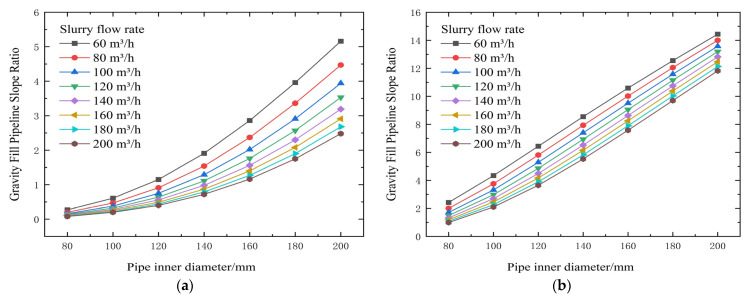

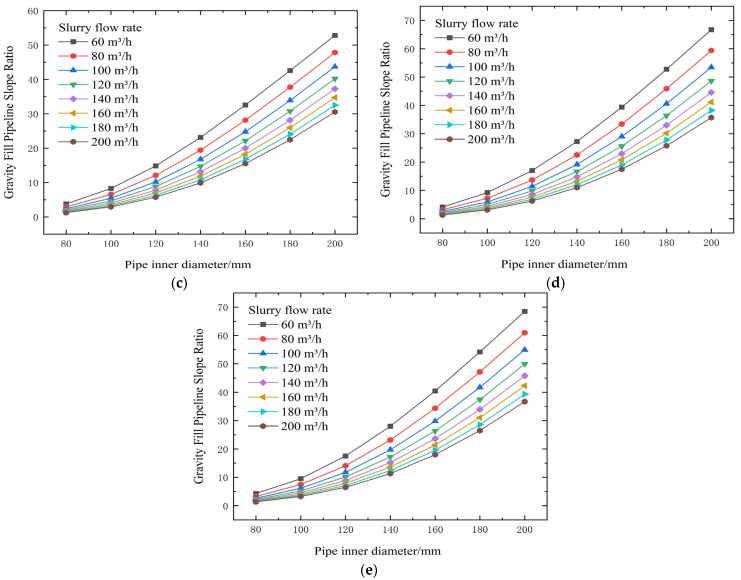
Calculated gravity backfill gradient for different concentrations, pipe diameters, and flow rates. (**a**) 74% slurry concentration, (**b**) 72% slurry concentration, (**c**) 70% slurry concentration (**d**) 68% slurry concentration, and (**e**) 66% slurry concentration.

From the test results and theoretical analysis in Table 7 and Figure 12 and Figure 13, both yield shear stress and viscosity coefficient increase sharply with increasing slurry concentration. When concentration decreases from 72% to 66%, yield stress decreases significantly from 59.24 Pa to 4.84 Pa, and the viscosity coefficient rapidly drops to around 0.720 Pa·s (example from context) after the concentration falls below 72%, thereby substantially reducing transport resistance. Increasing flow velocity significantly increases resistance. For a 120 mm pipe diameter, increasing the flow rate from 60 m^3^/h to 120 m^3^/h raises resistance from 12.64 kPa/m to 23.09 kPa/m, resulting in a backfill gradient below 1, making gravity flow difficult. Increasing the pipe diameter from 80 mm to 140 mm can drastically reduce transport resistance from 91.42 kPa/m to 11.28 kPa/m, correspondingly increasing the backfill gradient from 0.16 to 1.33, transforming the slurry from non-transportable to gravity-flow capable.

Notably, at a concentration of 74%, the slurry prepared with Baishushan PO42.5 cement exhibited a significantly higher yield stress of 59.24 Pa, whereas the unclassified tailings slurry showed a value of 35.19 Pa and slurries with other cement types ranged from 40.89 Pa to 45.70 Pa. This marked increase is likely attributed to the intrinsic physicochemical properties of the Baishushan PO42.5 binder. Specifically, variations in cement fineness characterized by specific surface area, along with gypsum content and clinker mineral composition, can accelerate early hydration kinetics. A faster initial hydration rate promotes the rapid formation of calcium silicate hydrate gels and ettringite, enhancing the flocculation structure of the fresh slurry and thereby increasing its yield stress, which represents the resistance to flow.

### 3.6. Selecting Cement Based on Technical and Economic Analysis

Through analysis, the average cost of cement materials for base grouting, one-step artificial pillar mining, two-step mining, and subsequent backfilling was used as a basis for comparison. The costs were as follows: Jinyuan PC32.5R cement: 69.60 yuan/m^3^; Kunlunshan PO42.5 cement: 67.65 yuan/m^3^; Baishushan PC32.5R cement: 94.14 yuan/m^3^; Baishushan PO42.5 cement: 68.92 yuan/m^3^. Kunlunshan PO42.5 cement had the lowest cost, while Baishushan PC32.5R cement had the highest cost. PO42.5 cement was significantly superior to PC32.5R cement in both technical and economic aspects. Comparing the PO42.5 cements from the two manufacturers, Kunlunshan PO42.5 cement had a slight economic advantage, with an average cement material cost of 1.28 yuan/m^3^ lower; Baishushan PO42.5 cement had a slightly greater technical advantage, requiring 12.2 kg less cement per m^3^ of backfill to achieve the same strength and offering a wider strength range under the same ash–sand ratio and concentration conditions. Both Kunlunshan PO42.5 cement and Baishushan PO42.5 cement are technically and economically sound cements, meeting the technical requirements and being suitable for use, but Kunlunshan PO42.5 cement is more cost-effective. Therefore, it is recommended to use Kunlunshan PO42.5 cement as the cementing agent for backfilling in this lead–zinc mine.

## 4. Discussion

### 4.1. The Mechanism of the Mutation Point at 72% Concentration

The results indicated a non-linear stepwise increase in yield stress, particularly when the concentration exceeded 72%. This phenomenon can be explained by the “water film thickness theory” and particle packing density. At lower concentrations, there is sufficient excess water to form a thick lubricating film around the tailings particles, resulting in low inter-particle friction. However, 72% represents a critical packing threshold for this specific unclassified tailings material. Beyond this point, the free water is rapidly converted into interstitial water, causing the water film to thin drastically. The direct contact probability between coarse particles increases, leading to a structural network formation that resists flow, which manifests as a sharp surge in yield stress.

The threshold of 72% solids concentration was derived empirically from the inflection points observed in the rheological data presented in Figure 3, Figure 4, Figure 5, Figure 6, Figure 7, Figure 8, Figure 9, Figure 10, Figure 11 and Figure 12 and theoretically supported by particle packing theory. As detailed in Table 4, Table 5, Table 6 and Table 7, while the absolute viscosity values vary with binder type due to differences in hydration kinetics and fineness, a consistent rheological transition is observed across all tested groups near this concentration. This threshold corresponds to the critical packing density of the unclassified tailings aggregate. Below 72%, the interstitial water volume is sufficient to form a lubricating film separating the particles, maintaining a low-viscosity regime regardless of the binder type tested. It should be noted that the current threshold is validated specifically for a cement-to-tailings ratio of 1:10 under ambient laboratory conditions. While the critical concentration is primarily governed by the tailings’ particle size distribution, extreme variations in binder dosage or ambient temperature could potentially shift this rheological boundary, which warrants further investigation.

### 4.2. Validity of the Simplified Pressure Drop Model

The pressure gradient calculation in this study utilizes a simplified solution based on the Bingham fluid model, neglecting the high-order term $\left[\frac{1}{3}(\tau_{0}/\tau_{w}{)}^{4}\right]$ in the Buckingham–Reiner equation. To explicitly quantify the error of this model compared to the theoretical exact solution, a rigorous sensitivity analysis was performed. Even under the most extreme experimental condition, where the yield stress is $\tau_{0}=35.19$ Pa and wall shear stress is $\tau_{w}\approx 109.35$ Pa, the calculated magnitude of the neglected term is merely $\frac{1}{3}(0.32{)}^{4}\approx 0.0035$. Consequently, the relative error introduced by this simplification is approximately 0.6%, which is significantly below the standard engineering tolerance of 5%. This negligible deviation demonstrates that the calculation model achieves high statistical accuracy and adequately represents the rheological behavior of the slurry with minimal computational error.

### 4.3. The Mechanisms Behind the Differences Between Different Types of Cement

Furthermore, the rheological divergence among different cements highlights the chemical–physical coupling effect. The Baishushan 42.5 cement exhibited significantly higher resistance than the others. This is distinct from previous studies that often treat cement merely as an inert filler in the fresh stage. Our findings suggest that for high-activity binders, the rapid dissolution of ions and the immediate formation of initial hydration products (e.g., ettringite) can bridge solid particles even within the short mixing duration, altering the effective particle size distribution and increasing the structural strength of the fresh slurry.

### 4.4. Transport Stability and Particle Suspension

Although the primary purpose of the L-shaped pipe experiment was not to conduct specific inlet and outlet particle size comparison tests, the stability of the slurry can be verified through the carrier fluid mechanism and rheological data. According to particle size analysis, fine particles smaller than 20 μm accounted for more than 15%. These fine particles, together with the mixing water, form a carrier fluid with viscosity and yield stress, which effectively suspends the coarse particles. This prevents particle segregation and stratification during gravity flow. Furthermore, the measured flow resistance remained stable throughout the simulation test and showed a consistent relationship with velocity (as shown in Figure 3 and Figure 5), indicating no accumulation of settled particles in the pipe. The yield shear stress calculated within the optimal concentration range of 66–74% (Table 4, Table 5, Table 6 and Table 7) was sufficient to overcome the gravitational force acting on the coarse particles, thus ensuring a uniform flow state.

### 4.5. Engineering Applications and Model Scalability

The model presented in this paper provides a general engineering framework for the design and optimization of backfill pipeline networks. By extracting intrinsic constitutive parameters, particularly the yield stress ($\tau_{0}$) and plastic viscosity (η), from standard L-shaped pipe tests, the model reliably scales rheological behavior from laboratory conditions to industrial applications. These material-specific parameters can be substituted into the resistance equation, replacing geometric variables with site-specific design constraints. This analytical approach allows for rigorous evaluation of hydraulic gradient requirements, enabling engineers to quantitatively determine the feasibility of gravity flow or specify pump capacity without relying on costly industrial-scale circulation experiments.

## 5. Conclusions and Outlook

### 5.1. Conclusions

Based on the multi-dimensional analysis of rheological behaviors, pipeline transport simulations, and cost-effectiveness evaluations, the following conclusions are drawn:

(1) Critical Rheological Threshold and Mechanism: A distinct rheological mutation point was identified at a mass concentration of 72%. This threshold corresponds to the critical packing density of the unclassified tailings. When the concentration exceeds 72%, the interstitial free water is exhausted, leading to direct particle-to-particle contact and an exponential increase in yield stress. Conversely, below 72%, the slurry maintains a stable “water film lubrication” state. Consequently, the optimal concentration range for gravity flow is determined to be 68–72%, which balances solids throughput with transport stability.

(2) Sensitivity of Transport Resistance: The flow resistance is highly sensitive to pipe diameter and slurry concentration. Theoretical calculations calibrated by L-pipe tests demonstrate that increasing the pipe diameter is the most effective method to reduce the hydraulic gradient. For instance, at a flow rate of $100\;m^{3}/h$, expanding the pipe diameter from 80 mm to 140 mm reduces the transport resistance by approximately 82%. This finding confirms that optimizing geometric parameters is crucial for deep-well backfill systems where gravity head is limited.

(3) Binder Selection and Chemical–Physical Coupling: The choice of binder significantly alters the fresh slurry rheology due to early-age hydration kinetics. Baishushan PO42.5 cement exhibited an anomalously high yield stress compared to other binders, attributed to its rapid formation of initial hydration products that enhance structural buildup. In contrast, Kunlun Mountain PO42.5 cement demonstrated a superior balance between fluidity and mechanical strength.

(4) Optimal Engineering Parameters: Through a comprehensive technical and economic analysis, Kunlun Mountain PO42.5 was selected as the optimal binder. The recommended industrial operation parameters are as follows: using unclassified tailings with Kunlun PO42.5 cement, controlling slurry concentration between 68% and 72%, and selecting a pipe diameter ≥120 mm to ensure a safety margin for gravity flow in deep mining environments.

### 5.2. Outlook

It should be noted that this study focuses on the macroscopic rheological and mechanical performance suitable for engineering design. Future research will incorporate microstructural analyses, such as SEM and XRD, to further elucidate the evolution of hydration products and the microscopic bonding mechanism of the cemented backfill.

Furthermore, based on the rheological parameter calculation model and the resistance evolution laws established in this study, the perspective of this work extends to the industrial upscaling of the theoretical model. While the current simplified model accurately predicts resistance in laminar flow, deep mining environments involve complex vertical-to-horizontal transitions where local turbulence may occur. Therefore, future work will utilize the obtained experimental rheological parameters ($\tau_{0},\eta$) and the identified optimal concentration range as input boundary conditions for Computational Fluid Dynamics simulations. This will enable the prediction of local resistance losses in complex pipe networks and facilitate the development of a ‘digital twin’ for the backfill pipeline system, bridging the gap between laboratory theoretical models and deep-well engineering applications.

Future work will focus on integrating non-contact monitoring technologies to validate the theoretical calculations presented in this study. Specifically, computer vision can be leveraged to track slurry level, surface velocity, and particle segregation along the pipeline in real time, offering independent verification of rheology and head-loss models. Robust and efficient vision-based models, such as DeepLab and EfficientNet, show significant promise for this application. DeepLab [39], a state-of-the-art semantic segmentation model, utilizes atrous convolution to capture multi-scale context, making it ideal for precisely delineating slurry boundaries and detecting sedimentation layers. Meanwhile, EfficientNet [40] proposes a compound scaling method that balances network depth, width, and resolution, providing a highly efficient architecture suitable for rapid, real-time visual recognition in industrial environments. Implementing these models would enhance the intelligence and reliability of backfill pipeline monitoring systems.

## Figures and Tables

**Table 1:** Chemical composition analysis results of tailings.

Chemical Component	O	Mg	Al	Si	S	K	Ca	Fe	Zn	Pb
**Content/%**	48	2.401	6.51	17.15	1.33	1.38	13.17	7.294	0.0958	0.0693

Note: O content estimated by stoichiometric balance.

**Table 2:** Basic physical parameters of unclassified tailings.

Test Item	Specific Gravity	Loose Dry Bulk Density (t/m^3^)	Compacted Dry Bulk Density (t/m^3^)	Loose Porosity (%)	Compacted Porosity (%)
**Data**	2.73	1.367	1.908	51.52	32.34

**Table 3:** L-pipeline simulation transport test results for unclassified tailings slurry.

No.	Mass Concentration (%)	Static Slurry Column Height (m)	Slurry Velocity (m/s)	Bulk Density (t/m^3^)	Slurry Unit Weight (N/m^3^)	Yield Shear Stress τ0 (Pa)	Shear Stress τ (Pa)	Viscosity Coefficient η (Pa·s)
1	74	0.30	0.68	1.883	18,450	35.19	109.35	0.693
2	72	0.18	1.52	1.839	18,020	22.61	99.66	0.343
3	70	0.065	2.48	1.797	17,610	8.08	83.03	0.219
4	68	0.044	2.86	1.757	17,220	5.40	73.64	0.174
5	66	0.4	3.01	1.719	16,850	4.81	68.88	0.156

**Table 4:** L-pipeline simulation transport test results for Jinyuan PC32.5R cement + tailings slurry.

Cement-to-Tailings Ratio	Mass Concentration (%)	Static Slurry Column Height (m)	Slurry Velocity (m/s)	Bulk Density (t/m^3^)	Slurry Unit Weight (N/m^3^)	Yield Shear Stress τ0 (Pa)	Shear Stress τ/Pa	Viscosity Coefficient η (Pa·s)
1:10	74	0.39	1.820	1.813	17,770	42.43	94.47	0.156
	72	0.081	2.565	1.774	17,390	9.87	80.33	0.196
	70	0.05	2.834	1.737	17,020	6.05	73.36	0.173
	68	0.035	3.013	1.701	16,670	4.18	68.11	0.156
	66	0.027	3.160	1.667	16,330	3.17	63.58	0.141

**Table 5:** L-pipeline simulation transport test results for Kunlun Mountain PO42.5 cement + tailings slurry.

Cement-to-Tailings Ratio	Mass Concentration (%)	Static Slurry Column Height (m)	Slurry Velocity (m/s)	Bulk Density (t/m^3^)	Slurry Unit Weight (N/m^3^)	Yield Shear Stress τ0 (Pa)	Shear Stress τ (Pa)	Viscosity Coefficient η (Pa·s)
1:10	74	0.35	1.144	1.892	18,540	40.89	106.52	0.341
	72	0.14	2.072	1.848	18,110	17.63	92.47	0.250
	70	0.045	2.698	1.805	17,690	5.67	79.09	0.199
	68	0.04	2.739	1.765	17,290	4.94	76.48	0.191
	66	0.034	3.512	1.726	16,910	4.12	57.35	0.111

**Table 6:** L-pipeline simulation transport test results for Baishushan PC32.5R cement + tailings slurry.

Cement-to-Tailings Ratio	Mass Concentration (%)	Static Slurry Column Height (m)	Slurry Velocity (m/s)	Bulk Density (t/m^3^)	Slurry Unit Weight (N/m^3^)	Yield Shear Stress τ0 (Pa)	Shear Stress τ (Pa)	Viscosity Coefficient η (Pa·s)
1:10	74	0.43	0.077	1.800	17,640	45.70	106.24	4.417
	72	0.32	2.217	1.762	17,270	34.83	85.91	0.134
	70	0.065	2.587	1.726	16,910	7.76	77.71	0.195
	68	0.048	2.844	1.691	16,570	5.66	71.20	0.168
	66	0.038	3.218	1.657	16,240	4.41	61.91	0.131

**Table 7:** L-pipeline simulation transport test results for Baishushan PO42.5 cement + tailings Slurry.

Cement-to-Tailings Ratio	Mass Concentration (%)	Static Slurry Column Height (m)	Slurry Velocity (m/s)	Bulk Density (t/m^3^)	Slurry Unit Weight (N/m^3^)	Yield Shear Stress τ0 (Pa)	Shear Stress τ (Pa)	Viscosity Coefficient η (Pa·s)
1:10	74	0.60	0.062	1.787	17,510	59.24	105.45	3.226
	72	0.33	1.550	1.749	17,140	35.04	94.47	0.231
	70	0.06	2.530	1.714	16,790	7.13	78.21	0.204
	68	0.043	2.678	1.679	16,460	5.05	73.94	0.188
	66	0.042	2.729	1.647	16,140	4.84	71.55	0.179

## Data Availability

The original contributions presented in the study are included in the article. Further inquiries can be directed to the corresponding author.
